# Cancer Stem Cell Markers for Urinary Carcinoma

**DOI:** 10.1155/2022/3611677

**Published:** 2022-03-15

**Authors:** Pu Xia, Da-Hua Liu, Ze-Jun Xu, Fu Ren

**Affiliations:** ^1^Biological Anthropology Institute, Liaoning Medical University, Jinzhou, Liaoning, China; ^2^Shenyang Medical College, Shenyang, Liaoning, China

## Abstract

Cancer stem cell (CSC) refers to cancer cells with stem cell properties, that is, they have the ability of “self-renewal” and “differentiation.” Cancer stem cells exist in cancer cells and are the “culprit” of cancer recurrence and metastasis. It is difficult to be found because of its small amount, and it is difficult for anticancer drugs to produce effects on it. At present, the isolation and identification of cancer stem cells from many solid tumors are still quite difficult, mainly due to the lack of specific molecular markers of cancer stem cells. In this review, cancer stem cell surface markers and functional markers in urinary system were summarized. These markers can provide molecular targets for cancer therapy.

## 1. Introduction

Great progress has been made in the treatment of cancer; however, the treatment is even less ideal [1]. In addition to local operation, the traditional radiotherapy and chemotherapy can hardly cure urinary carcinoma [2]. The increasingly recognized complexity of human cancers and the heterogeneity of malignant tumor cells have posed a major challenge to the development of effective therapies in urinary carcinoma [3]. A small group of stem cell-like tumor cells has been found in tumor tissue [4]. The cells have the potential of self-renewal, unlimited proliferation, and multidirectional differentiation [4]. Although the number is small, they play an important role in tumor formation, recurrence, and metastasis [5]. These cells are called cancer stem cells or tumor-initiating cells [6]. Cancer stem cells have their own characteristics: (1) self-renewal ability: cancer stem cells are produced by symmetric division to complete self-replication; (2) differentiation potential: generation of tumor cells with different degrees of differentiation through asymmetric division; (3) heterogeneity: functional heterogeneity among cancer stem cells [6].

The discovery of cancer stem cells has undoubtedly opened up a new way to better understand tumor biology and explore new methods for cancer treatment. However, until now, no one knows which cells are cancer stem cells. The cells which have stem cell features can be separated and purified by flow cytometry (FACS) and magnetic-activated cell sorting (MACS) by surface markers and functional markers [7]. Among these markers, which are the specific markers of urologic oncology stem cells are still controversial. In this paper, we will review the widely studied and newly discovered tumor markers in urologic oncology stem cells, so as to provide targets for the radical treatment of urologic oncology.

## 2. Prostate Cancer

**Figure 1:**
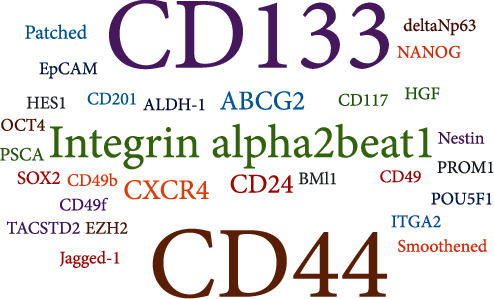
Word cloud: visualization of the surface markers for prostate cancer.

Prostatic epithelial cells which consist of two layers of epithelial cells, basal cells, glandular epithelial cells, and neuroendocrine cells are a complex tissue in structure and function [8]. In addition, there is a small group of special types of cells with self-renewal ability and differentiation potential, so they are called “prostate stem cells” [8]. The cells express cytokeratin 5 and 19 (CK5, CK19), androgen receptor (AR), prostate-specific antigen (PSA), and prostatic acid phosphatase (PAP) [8]. The origin of prostate cancer stem cells is still controversial. Prostate cancer stem cells are derived from normal stem cells, transient expanded epithelial cells, or terminal differentiated coelomocytes [9]. Prostate cancer stem cell markers can provide targets for the treatment of prostate cancer [10]. At present, some prostate cancer stem cell markers such as CD44 and CD133 have been widely studied (Figure 1). Some new markers have been found, such as integrin *α*2, *α*6, *β*1, c-Met, aldehyde dehydrogenase 1 (ALDH1), ABCG2, CD166, Sox2, and EZH2 (Figure 1).

### 2.1. CD44

CD44, a cell surface protein, plays a role in cell adhesion and signal transduction [11]. It has been identified as a surface marker for prostate cancer stem cells [12]. CD44^+^ cells obtained from prostate transplantation tumor have tumorigenicity, clonogenic capacity, and metastatic potential [13]. CD44^+^ cells from LnCAP, PC3, and DU145 cells have stronger proliferation and tumorigenesis than CD44^−^ cells [14]. The genes related to the proliferation, regeneration, and differentiation of stem cells, such as Sox2, OCT3/4, SMO, and *β*-catenin, were highly expressed in CD44^+^ prostate cancer cell [15].

### 2.2. CD133

CD133 is a glycoprotein with five transmembrane domains, which is expressed in different types of stem cells and endothelial progenitor cells [16]. CD133 has been used to isolate cancer stem cells in many cancer types, including prostate cancer [17]. CD133^+^ cells derived from prostate cancer tissue showed more proliferative and aggressive than CD133^−^ cells [18]. These cells have similar phenotype with normal prostate stem cells [18]. Both CD133^+^ prostate cancer stem cells and normal prostate stem cells express basal cell markers, but not androgen receptors (AR) [18]. In addition, the tumorigenicity of CD133^+^ cells from DU145 cells was similar to that of CD133^−^ ones [18]. Based on these evidences, CD133 may be a not suitable stem cell marker for prostate cancer. Interestingly, CD44^+^/*α*2*β*1^hi^/CD133^+^ prostate cancer cells have self-renewal ability and can differentiate into prostate cells that can express AR and PAP [19]. In the case of doubts about CD133, more specific prostate cancer stem cell markers need to be used with CD133 to target prostate cancer stem cells.

### 2.3. Aldehyde Dehydrogenase 1 (ALDH1)

ALDH1, expressed widely in normal tissues, has been used as a functional marker for cancer stem cells [20–22]. It can convert aldehydes into carboxylic acids and participate in the degradation of intracellular toxic substances and cell protection [23]. Compared with normal prostate tissues, ALDH1 expressed higher in prostate cancer tissues [24, 25]. High expressed ALDH1 level was associated with the higher Gleason score, the higher the pathological grade, and the lower overall survival rate of prostate cancer patients [24, 25]. ALDH1^high^ murine prostate stem cells have high proliferation potential *in vitro* and more effective in generating prostatic tissue *in vivo* [26]. ALDH1 may act as detoxification enzymes to protect stem cells from toxic compounds [27]. If ALDH1 can be effectively inhibited, prostate cancer stem cells will not escape the attack of traditional chemoradiotherapy.

### 2.4. ATP Binding Membrane Transporter (ABCG2)

ABCG2 is an ATP binding membrane transporter, which can pump antitumor drugs out of cancer cells and protect the cells from killing [28]. ABCG2 is also associated with multidrug resistance of prostate cancer [29]. The cells, enriched by ABCG2, were called side population (SP) cells [29]. SP cells have the characteristics of cancer stem cells, such as drug resistance [30]. ABCG2^+^/AR^+^ prostate cancer stem cells can survive under castration, chemotherapy, and hypoxia environment [31]. So, ABCG2 can be used as a functional marker to target prostate cancer stem cells. However, Patrawala et al. [32] found that ABCG2^+^ cells and ABCG2^−^ cells from DU145 cells have similar tumorigenicity. Some ABCG2^−^ DU145 cells also have the ability to generate ABCG2^+^ ones [32]. ABCG2-mediated androgen efflux decreased the nuclear AR expression and induced cell growth in HPr-1-AR (nontumorigenic) and CWR-R1 (tumorigenic) prostate cell lines [31]. It seems that ABCG2 as a specific marker of prostate cancer stem cells is still controversial. However, all this evidence indicated that ABCG2 maintains the characteristics of AR^+^ prostate cancer stem cells.

### 2.5. Integrin *α*2*β*1

Integrin is a kind of collagen receptor or extracellular matrix protein, which plays an important role in cell survival, proliferation, and metastasis [33]. The growth and metastasis of prostate cancer were closely related to the expression of integrin [34]. Goel et al. [35] found that most *α* and *β* integrins were out of control in prostate cancer. The expression of *α*2 in lymph node metastasis was higher than that in primary tumor [35]. *β*1 expressed higher in the high tumor grade [35]. Both prostate cancer cells and prostate cancer stem cells express high levels of Integrin *α*2*β*1 [36]. DU145 and PC3 cells with docetaxel treatment expressed significantly higher *α*2*β*1 levels than untreated ones [36, 37]. *α*2*β*1^high^ DU145 and PC3 cells were more invasive than *α*2*β*1^low^ ones [36, 37].

### 2.6. CXCR4

CXCR4 is a GPCR (G-protein coupled receptor) composed of 352 amino acids and has seven transmembrane structures [38]. CXCR4 is mainly expressed in embryonic stem cells, hematopoietic stem cells, endothelial stem cells, and other pluripotent stem cells [39]. CXCR4 is involved in a variety of physiological mechanisms *in vivo*, including HIV-1 virus infection [40], hematopoietic function [41], embryonic development [42], and tumor migration [43]. CXCR4 is a specific receptor for chemokine stromal cell-derived factor-1 (CXCL12) [44]. In our previous study, we confirmed the CXCR4/CXCL12 axis regulates self-renew, differentiation, and tumorigenicity of DU145 and PC3 cells [45]. CXCR4 and CD133 coexpressed in prostate epithelial cells and cancer cells of patients specimens [46]. CXCR4 induced a more aggressive phenotype in prostate cancer [47].

### 2.7. Sox2, EZH2, and Oct4

Both sex-determining region Y-box 2 (Sox2) and enhancer of zeste homolog 2 (EZH2) play important roles in the development of human embryonic stem cells [48, 49]. Sox2 is a transcription factor, which plays a key role in maintaining the self-renewal and undifferentiated state of embryonic stem cells [48]. EZH2 is necessary for the in vitro reconstruction of embryonic stem cells and plays a key role in embryonic formation [49]. Sox2 and EZH2 also play a key role in prostate cancer stem cells (PCSCs). Ugolkov et al. [50] found that EZH2 and Sox2 were closely related in prostate cancer, and almost 90% Sox2^+^ prostate cancer was EZH2^+^ type by immunohistochemistry and tissue microarray. Oct4, also referred to as POUF51 and Oct3/4, works synergistically with Sox2 for maintaining pluripotency of cells [51]. Although Sox2, EZH2, and Oct4 are not used as prostate cancer stem cell markers, more and more studies confirmed the regulatory roles of these genes in PCSC. EZH2 was upregulated in PCSCs compared with non-PCSCs [52]. A recent study in our group showed that inhibition of EZH2-mediated histone methylation improves the radiotherapy for PCSC [53]. Sox2 regulates self-renewal and anchorage-independent growth of DU145 spheres depending on EGFR expression [54]. Sox2/Oct4 overexpression in PCSCs contributes to tumor initiation and progression [55]. Sox2/Oct4 positive cells that isolated from prostate cancer tissues have the stem cell properties [55].

## 3. Bladder Cancer

**Figure 2:**
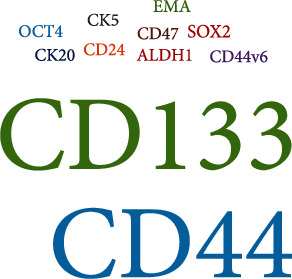
Word cloud: visualization of the surface markers for bladder cancer.

The basal cell layer of normal bladder epithelium contains undifferentiated cells [56]. These cells move to the surface of bladder cavity gradually and complete the differentiation in this process [56]. In view of the this reason, many studies on bladder cancer stem cells focus on bladder regenerative basal cell layer. CD44, CD133, and other markers are expressed in both the basal cell layer and bladder cancer stem cells (Figure 2).

### 3.1. CD44

McKenney et al. [57] found CD44^+^ cells in the basal cell layer of normal urothelial epithelium and urothelial carcinoma. Chan et al. [58] isolated and identified a subpopulation of primary bladder cancer cells based on CD44^+^, CK5^+^, and CK20^−^ and further confirmed that this subpopulation can induce tumorigenesis in nude mice. In addition, the tumorigenicity of CD44^+^ cells was 10-200 times higher than that of CD44^−^ ones from nude mice [58]. In bladder cancer tissue, epithelial membrane antigen (EMA) negative CD44 variant subtype (CD44v6) positive cells account for about 30% of cancer cells [59]. These cells have a higher self-renewal ability compared with parental tumors cells [59]. Therefore, tumor initiation ability of CD44^+^ cells is confirmed, and CD44 can be used as a sorting marker of bladder cancer stem cells (BCSCs).

### 3.2. CD133

Zhu et al. [60] demonstrated that CD133 was highly expressed in mouse bladder cancer cells (MCSCs) by flow cytometry (FACS) and quantitative PCR. Bentivegna et al. [61] isolated CD133^+^ cells by immunofluorescence analysis and cytogenetic analysis in 49 samples of bladder cancer tissues. The cells were identified as BCSCs by transplantation in nude mice [61]. Huang et al. [62] demonstrated the stem cell-like characteristics of CD133^+^ subpopulation of human bladder cancer cell J82. All this evidence showed that CD133 can also be used as surface markers for sorting BCSCs.

### 3.3. CD47

CD47, also known as integrin-associated protein (IAP), exists on the cell surface and belongs to the transmembrane glycoprotein of immunoglobulin superfamily [63]. The expression of CD47 is related to tumor immunity and prognosis [64]. CD47 is widely expressed in bladder cancer cells [65], however, expressed higher in BCSCs than other cancer cells [66]. CD47 could bind to the macrophage surface receptor SIRP*α* and block macrophage phagocytosis [67]. CD47 may be a target for bladder cancer immunotherapy and a BCSC marker.

### 3.4. ALDH1A1

ALDH1A1 is a member of aldehyde dehydrogenase gene families [68]. ALDH1A1^+^ bladder cancer cells had higher tumorigenicity than ALDH1A1^−^ cancer cells both *in vivo* and *in vitro* [69, 70]. The ALDH1A1^+^ bladder cancer cells can represent CSCs in bladder cancer. Bladder cancer patients with the high expression of ALDH1A1 were significantly associated with poor prognosis [71].

### 3.5. Cytokeratin

Cytokeratin (CK) is a major skeletal protein, which exists in keratinocytes of epithelial cells [72]. The expression of CK reflects cell differentiation and can be used as a common tumor diagnostic marker [73]. As mentioned above, CD44^+^CK5^+^CK20^−^ bladder cancer cells were BCSCs; CK20 was not expressed in these cells [58]. CK17 exists in urothelial basal cells and has strong tumorigenicity [74]. He et al. [74] confirmed that CK17^+^/CK18^−^/CK20^−^ could be used for isolation and identification BCSCs. CK20, CK17, and CK18 may be considered as markers related to BCSCs.

### 3.6. Oct3/4

OCT4 is a key regulator of tumor progression, aggressive behavior, and metastasis of bladder cancer [75]. Atlasi et al. [76] found that bladder transitional cell carcinoma with high Oct3/4 expression has faster tumor progression and shorter tumor-related survival time than superficial bladder transitional cell carcinoma with medium and low expression of Oct3/4. It maintains self-renewal and differentiation of bladder cancer cells [76]. Bentivegna et al. [61] isolated BCSCs with high expression of Oct4 from the tissues of patients with bladder cancer and proved the stem cell-like properties of these cells. Oct4 may lead to tumorigenesis and is closely related to the treatment resistance or recurrence of bladder cancer [61].

## 4. Renal Carcinoma

**Figure 3:**
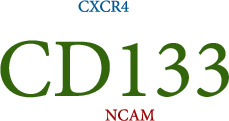
Word cloud: visualization of the surface markers for renal carcinoma.

Compared with other urological tumors, the research of renal cancer stem cells is later [77]. Although some markers have been studied (Figure 3), whether these markers can be used as specific surface markers for identification of renal cancer stem cells still needs to be confirmed in subsequent experiments.

### 4.1. CD133

CD133^+^ renal adult stem cells in human renal cortex have proliferation and self-renewal ability and can differentiate into renal tubular epithelial cells and vascular endothelial cells [78]. CD24^+^CD133^+^ epithelial cells isolated from glomerular capsule have the potential to differentiate into osteocytes, adipocytes, and nerve cells *in vitro* and can induce the formation of renal tubular structure *in vivo* [79]. CD133^+^ cells were involved in the formation of new blood vessels in renal cell carcinoma, but it is not clear whether these markers can be used to isolate and identify renal cancer stem cells [80].

### 4.2. CXCR4

CXCR4 is an important marker of renal cell carcinoma stem cells [81]. The number of renal cancer stem cells and the expression of CXCR4 increased with the increase of malignancy of RCC [81]. The cancer cells isolated from patients with stage IV renal carcinoma (RCC) had more obvious characteristics of tumor stem cells than those from patients with stage I RCC [81]. CXCR4 was expressed in both types of cells [79]. In the experiment of tumor formation, stage IV RCC cells were more likely to form tumors than stage I RCC cells [81]. Compared with other markers, CXCR4 is considered to be the most reliable stem cell marker of RCC.

## 5. Summary

Cancer stem cell theory brings a revolutionary change to cancer research. It has promoted the exploration of biological characteristics and molecular mechanism of cancer cells and provided a new clear and reliable direction for the understanding of the origin and the clinical treatment of cancer. The research of urological cancer stem cells started relatively late, facing great opportunities and challenges. If the specific surface markers of cancer stem cells can be identified, drugs targeting cancer stem cells will also be developed and applied, and the cure of cancer will not be just a dream in people's hearts.
